# Risk factors for chemotherapy‐induced peripheral neuropathy in patients receiving taxane‐ and platinum‐based chemotherapy

**DOI:** 10.1002/brb3.1312

**Published:** 2019-05-07

**Authors:** Alex Molassiotis, Hui Lin Cheng, Kwun To Leung, Yu Chung Li, Kam Hung Wong, Joseph Siu Kie Au, Raghav Sundar, Alexandre Chan, Terrence Rong De Ng, Lorna K. P. Suen, Choi Wan Chan, Janelle Yorke, Violeta Lopez

**Affiliations:** ^1^ School of Nursing The Hong Kong Polytechnic University Hong Kong Hong Kong SAR; ^2^ Department of Clinical Oncology Queen Elisabeth Hospital Hong Kong Hong Kong SAR; ^3^ Department of Oncology Hong Kong Adventist Hospital Hong Kong Hong Kong SAR; ^4^ Department of Haematology‐Oncology National University Health System Singapore; ^5^ Department of Pharmacy National University of Singapore Singapore; ^6^ Division of Nursing, Midwifery & Social Work University of Manchester Manchester UK; ^7^ Alice Lee Centre for Nursing Studies National University of Singapore Singapore

**Keywords:** cancer, chemotherapy‐induced peripheral neuropathy, platinum chemotherapy, risk factors, taxanes

## Abstract

**Background:**

Chemotherapy‐induced peripheral neuropathy (CIPN) is a significant and difficult to manage side effect of neurotoxic chemotherapies. Several risk factors for CIPN have been identified to date, but inconsistencies and methodological limitations exist in past research. Also, a limited number of potential risk factors has been investigated in the past.

**Aim:**

The objective of this study was to assess the relative contribution of a wider range of risk factors in the development of CIPN.

**Methods:**

This analysis used the 6‐month data after starting chemotherapy from a larger prospective observational study on CIPN risk, prevalence, and quality of life. Patients were assessed at recruitment for possible CIPN risk factors, including prior history of neuropathies, current/past infectious diseases; neurotoxic medication history; personal and treatment characteristics; smoking history, alcohol use, and vegetable/fruit intake. Neuropathy was assessed at 6‐months after starting chemotherapy with the neuropathy (motor/sensory) items of the NCI‐CTCAE scale and the WHO criterion for neuropathy. Data on symptom burden were also collected.

**Results:**

Data were available from 255 patients from three cancer centers in Hong Kong, Singapore, and UK. The use of different scales did not always identify the same predictor variables. Key risk factors in multivariate regression models included older age (highest OR = 1.08, *p* < 0.01 with the WHO scale), chemotherapy (platinum‐based chemotherapy had OR = 0.20–0.27 in developing CIPN compared to taxane‐based chemotherapy), history of neuropathy (for motor CIPN only, OR = 8.36, *p* < 0.01), symptom burden (OR = 1.06, *p* < 0.05), number of chemotherapy cycles received (OR = 1.19–1.24, *p* < 0.01), and alcohol intake (OR = 0.32, *p* < 0.05). In univariate analysis, the use of statins was implicated with CIPN (*p* = 0.03–0.04 with different assessments) and diabetes showed a trend (*p* = 0.09) in the development of CIPN.

**Conclusion:**

This study confirmed the CIPN risk related to certain variables and identified new ones. This knowledge can assist with treatment decisions and patient education.

## 1 INTRODUCTION

Chemotherapy‐induced peripheral neuropathy (CIPN) is a common side effect of taxane‐ and platinum‐based chemotherapy, with prevalence ranging from 12%–96% (Eckhoff, Knoop, Jensen, & Ewertz, 2015; Osmani et al., 2012; Seretny et al., 2014). The impact of CIPN on patients’ quality of life can be significant (Ezendam et al., 2014; Mols, Beijers, Vreugdenhil, & Poll‐Franse, 2014; Seretny et al., 2014). CIPN may be challenging for clinicians to diagnose, assess and manage, especially in patients with co‐existing or preexisting conditions or disorders that involve the peripheral nervous system (Hausheer, Schilsky, Bain, Berghorn, & Lieberman, 2006). A general predisposition for developing CIPN is observed in nerves previously damaged by diabetes mellitus, alcohol, or inherited neuropathy (Quasthoff & Hartung, 2002). Thyroid dysfunction, metabolic and infectious diseases (i.e., hepatitis B or C, poliomyelitis, HIV), vitamin deficiencies (i.e., B12, B1, B6), and monoclonal gammopathy have also been implicated in the pathogenesis of CIPN (Armstrong, Almadrones, & Gilbert, 2005; Kaley & DeAngelis, 2009). Many medications that are commonly used in cancer patients, such as metronidazole, misonidazole, sulfasalazine, or phenytoin, are all documented to be associated with some degree of peripheral neurotoxicity (Hausheer et al., 2006).

Research around risk factors for CIPN has increased over recent years, although at times findings are inconsistent or a limited pool of potential factors is assessed. In a large study (*n* = 3,106), worse neurotoxicity was observed in colorectal cancer patients, those with longer duration of cancer, on current therapy, older patients, and in African Americans (Lewis et al., 2015). Being obese and having more insomnia severity, anxiety, and depression were all associated with CIPN in other studies (Bao et al., 2016; Simon, Danso, Alberico, Basch, & Bennett, 2017). Older age, lower income, higher BMI, comorbidities, being born prematurely, higher cumulative dose of chemotherapy, and poorer functional status were also predictive of CIPN (Miaskowski et al., 2017). Diabetes was also shown to be predictor of CIPN (Ottaiano et al., 2016) although other studies have found no such link (Pereira et al., 2016; Simon et al., 2017). However, many of the potential predictors of CIPN have not been fully investigated to date. Hence, the aim of this study was to assess the relative contribution of a wider range of risk factors in the development of CIPN, providing a stronger explanatory model, and further explore the potential link between CIPN and other symptoms.

## 2 MATERIALS AND METHODS

### 2.1 Design

This analysis used data from the 6‐month CIPN assessment after starting chemotherapy from a larger prospective observational study on CIPN prevalence and quality of life (Molassiotis et al., 2019), focusing on one of the primary objectives of the study.

### 2.2 Sample and settings

The sample included patients receiving platinum‐based chemotherapy (primarily cisplatin) and taxane‐based chemotherapy (primarily docetaxel) for the treatment of breast, lung, ovarian, gastrointestinal, head & neck as well as urinary tract cancers. Data were collected from specialist oncology clinics in three countries/regions (Hong Kong, Singapore, and Manchester in the UK). The study was approved by the ethics committees of the Hong Kong Polytechnic University, Hong Kong; Central Cluster of the Hospital Authority, Hong Kong; The National University Hospital; Singapore; The University of Manchester, Manchester, UK; and the Central Manchester Research and Ethics Committee. All participants have provided written informed consent.

## 3 PROCEDURES

Eligible patients were identified and approached at hospital outpatients clinics. Those who agreed to participate and provided informed signed consent completed all the baseline measurements including personal characteristics and presence of potential risk factors as identified in the literature. Clinical data were obtained from the medical records as well as information on medication used and past medical history. Participants in the larger project underwent a neuropathy assessment repeated at each cycle of chemotherapy (up to six cycles), 6 months, 9 months, and 12 months postchemotherapy. For the current analysis, data from the 6‐month assessment were used as it had the highest number of patients across all assessments and the highest incidence of CIPN. Ethical approval was obtained from each site before commencing the study.

## 4 OUTCOME MEASURES

### 4.1 Risk assessment

Potential risk factors identified previously in the literature (Armstrong et al., 2005; Hauseer et al., 2006; Kaley & DeAngelis, 2009; Miaskowski et al., 2017; Ottaiano et al., 2016; Quasthoff & Hartung, 2002) were examined for their impact in the development of CIPN. These included:

- Diagnosis with acquired or hereditary neuropathy such as diabetes, renal disease, hypothyroidism, connective tissue disease. Prior history of neuropathy or family history of neuropathy; vitamin deficiencies (Hershman et al., 2016; Ottaiano et al., 2016; Seretny et al., 2014).
- Diagnosis with current or previous infectious diseases (HIV; Poliomyelitis; Hepatitis B or C; Armstrong et al., 2005; Kaley & DeAngelis, 2009; Seretny et al., 2014).
- Neurotoxic medication history (a list of 51 medications linked with neurotoxicity, such as cyclosporine, vancomycin, cimetidine, etc; Hauseer et al., 2006; Kaley & DeAngelis, 2009; Quasthoff & Hartung, 2002).
- Personal and treatment characteristics:
  o Age (Lewis et al., 2015; Miaskowski et al., 2017, 2018).
  o Disease site (Quasthoff & Hartung, 2002; Seretny et al., 2014; Simon et al., 2017).
  o Chemotherapy type (taxanes; platinum‐based chemotherapy; combination of taxanes and platinum‐based chemotherapy), number of chemotherapy cycles, and cumulative dosage of each neurotoxic chemotherapy drug (Kaley & DeAngelis, 2009; Quasthoff & Hartung, 2002; Simon et al., 2017).
  o Smoking history (never smoked; current smoker; ex‐smoker; Kawakami et al., 2012; Seretny et al., 2014).
  o History of alcohol intake (Pereira et al., 2016) (drinks per day (number) using an explanatory diagram on quantity (i.e., small glass of wine (120 ml) = 1 drink, etc).
  o Dietary history (servings of fruits and vegetables per day with explanations, i.e., 1 serve = 1 fruit) (Greenlee et al., 2016).

### 4.2 NEUROTOXICITY ASSESSMENT

1. The National Cancer Institute – Common Terminology Criteria for Adverse Events (NCI‐CTCAE) version 4.03 is a physician‐rated grading system that includes criteria and definitions for quantifying and grading CIPN. This grading scale comprises two items, with a sensory and a motor assessment and utilizes a 5‐point scale ranging from grade 1 to grade 5.
2. The WHO criterion is also a physician‐rated CIPN item, and includes paresthesia, reflex decreases and extend of motor loss as parameters (WHO, 1979).

These two assessments were completed using both a checklist of neuropathy‐related indications and physical/neurological examination to aid in the diagnosis. Also, a new composite variable (combined scale, supported by the combined scale's Cronbach alpha of 0.74, intraclass correlation of 0.74 and item‐to‐item correlations of 0.41–0.61, *p* < 0.01) was also created with a combination of the above three items, in order to have maximum variation in the data, as the two scales were identifying varying prevalence of CIPN at different patients (the highest prevalence rate with the WHO criterion item). This combined outcome variable was flagged as having CIPN when at least one of the three items used in the assessment of CIPN indicated so, and it was used in the risk factor analysis.

### 4.3 Symptom burden

This variable responds to a secondary objective of the study to explore any links between CIPN and other symptoms. In order to estimate symptom burden, we used data from the single‐item symptom measures (items 8, 9, 11–25 of the European Organization for Research and Treatment (EORTC) QLQ‐C30. It incorporates nine multi‐item scales to assess quality of life: five functional scales (physical, role, cognitive, emotional, and social); three symptom scales (fatigue, pain, and nausea and vomiting); and a global health and quality‐of‐life scale (Aaronson et al., 1993). In order to estimate symptom burden, we used data from the single‐item symptom measures (items 8,9, 11–25 of the scale, including pain, tiredness, appetite loss, breathlessness, nausea, vomiting, constipation, diarrhea, cognitive impairment, psychological symptoms) after transforming them to 0–100 scores, thus creating a new predictor variable of “symptom burden”. This scale has been validated in China (Wan et al., 2008) and Singapore (Tan et al., 2014). Its Cronbach's alpha in our sample was 0.90.

## 5 DATA ANALYSIS

Descriptive statistics were used to summarize the data. Chi‐square analysis assessed differences in categorical variables while Student's *t* tests were used for comparing continuous variables. Logistic regression models were used for the main risk factor analysis. The relevant covariates for initial model inclusion were identified using a multivariate analysis, with rules (*p*‐values < 0.20) for retaining variables in the model. This was followed by the final model which only included significant (defined above) variables. This is a recommended approach for removing unimportant covariates so that a more manageable set of variables can be used with more complex multivariate statistical techniques (Lee, 2014). A multilevel logistic regression analyses took place taking account of center effect and time since last cycle of chemotherapy, to develop the predictive model for CIPN. Data were analyzed using SPSS v.21.

## 6 RESULTS

### 6.1 Sample characteristics

Data from 255 participants were available for analysis at the 6‐month assessment of CIPN (chosen as this point had the highest CIPN rate and highest number of participants). The larger study had 343 patients at baseline and 2,399 observations in total, although numbers decreased over time due to patients discontinuing chemotherapy, patient death, or relocation of patients. There were 162 participants from Hong Kong (63.5%), 78 from Singapore (30.6%), and 15 from the UK (5.9%). The majority were breast cancer patients followed by lung cancer and gynecological cancer patients, receiving adjuvant chemotherapy, and at stage II or III of their cancer. Sample characteristics are shown in Tables 1 and 2. Analysis with individual chemotherapy types (i.e., docetaxel or cisplatin) was done initially separately, and as common risk factor patterns were observed across protocols, the whole data was subsequently analyzed and reported together.

**Table 1:** Chemotherapy‐induced peripheral neuropathy and its risk factors in categorical variables (*n* = 255)

Variable	Frequency	Chemotherapy‐induced peripheral neuropathy (CIPN) frequency and percentage in each scale			
		CTCAE‐motor	CTCAE‐sensory	WHO item	Combined CIPN scales
Overall	255 (100%)	36 (14.1%)	33 (12.9%)	45 (17.6%)	68 (26.7%)
Race					
Chinese	210 (82.4%)	30 (14.3%)	26 (12.4%)	34 (16.2%)	55 (26.2%)
Non‐Chinese Asians	23 (9.0%)	3 (13.0%)	4 (17.4%)	9 (39.1%)	9 (39.1%)
Caucasian	22 (8.6%)	3 (13.6%)	3 (13.6%)	2 (9.1%)	4 (18.2%)
*p‐value*		0.98	0.79	0.01	0.26
Chemotherapy group					
Taxanes	123 (48.2%)	25 (20.3%)	24 (19.5%)	30 (24.4%)	46 (37.4%)
Platinum	64 (25.1%)	4 (6.3%)	3 (4.7%)	6 (9.4%)	7 (10.9%)
Combined	68 (26.7%)	7 (10.3%)	6 (8.8%)	9 (13.2%)	15 (22.1%)
*p‐value*		0.02	0.008	0.02	<0.001
Treatment intent					
Radical (adjuvant)	157 (61.6%)	24 (15.3%)	19 (12.1%)	22 (14.0%)	39 (24.8%)
Radical (neoadjuvant)	43 (16.9%)	6 (14.0%)	7 (16.3%)	10 (23.3%)	14 (32.6%)
Radical (concurrent)	14 (5.5%)	1 (7.1%)	1 (7.1%)	0 (0.0%)	1 (7.1%)
Palliative	41 (16.1%)	5 (12.2%)	6 (14.6%)	13 (31.7%)	14 (34.1%)
*p‐value*		0.83	0.79	0.01	0.18
Chemotherapy protocol					
Paclitaxel	27 (10.6%)	14 (51.9%)	15 (55.6%)	14 (51.9%)	15 (55.6%)
Docetaxel	96 (37.6%)	16 (16.7%)	31 (32.3%)	16 (16.7%)	31 (32.3%)
Cisplatin	41 (16.1%)	2 (4.9%)	3 (7.3%)	2 (4.9%)	3 (7.3%)
Oxaliplatin	20 (7.8%)	4 (20.0%)	4 (20.0%)	4 (20.0%)	4 (20.0%)
Carboplatin and docetaxel	28 (11.0%)	2 (7.1%)	3 (10.7%)	2 (7.1%)	3 (10.7%)
Carboplatin and paclitaxel	34 (13.3%)	7 (20.6%)	12 (35.3%)	7 (20.6%)	12 (35.3%)
*p*‐value		0.13	0.03	<0.001	<0.001
Diagnosis					
Ovarian	25 (9.8%)	4 (16.0%)	4 (16.0%)	3 (12.0%)	7 (28.0%)
Lung	28 (11.0%)	1 (3.6%)	1 (3.6%)	1 (3.6%)	1 (3.6%)
Head and neck	17 (6.7%)	1 (5.9%)	1 (5.9%)	1 (5.9%)	2 (11.8%)
Breast	146 (57.3%)	25 (17.1%)	23 (15.8%)	32 (21.9%)	49 (33.6%)
Colorectal	15 (5.9%)	0 (0.0%)	0 (0.0%)	3 (20.0%)	0 (0.0%)
Others	24 (9.4%)	5 (20.8%)	4 (16.7%)	5 (20.8%)	5 (20.8%)
*p‐value*		0.15	0.25	0.15	0.02
Stage					
I	41 (16.1%)	6 (14.6%)	5 (12.2%)	3 (7.3%)	9 (22.0%)
II	79 (31.0%)	14 (17.7%)	10 (12.7%)	17 (21.5%)	24 (30.4%)
III	84 (32.9%)	8 (9.5%)	9 (10.7%)	11 (13.1%)	18 (21.4%)
IV	51 (20.0%)	8 (15.7%)	9 (17.6%)	14 (27.5%)	17 (33.3%)
*p‐value*		0.49	0.71	0.04	0.34
Metronidazole	12 (4.7%)	3 (25.0%)	2 (16.7%)	3 (25.0%)	5 (41.7%)
*p‐value*		0.23	0.48	0.45	0.23
Statins	37 (14.5%)	7 (18.9%)	9 (24.3%)	9 (24.3%)	15 (40.5%)
*p‐value*		0.25	0.03	0.25	0.04
Gender					
Male	49 (19.2%)	4 (8.2%)	3 (6.1%)	8 (16.3%)	9 (18.4%)
Female	206 (80.8%)	32 (15.5%)	30 (14.6%)	37 (18.0%)	59 (28.6%)
*p‐value*		0.18	0.11	0.79	0.14
Smoking history					
Never	199 (78.0%)	29 (14.6%)	27 (13.6%)	36 (18.1%)	57 (28.6%)
Current	7 (2.7%)	1 (14.3%)	1 (14.3%)	3 (42.9%)	3 (42.9%)
Ex‐smoker	49 (19.2%)	6 (12.2%)	5 (10.2%)	6 (12.2%)	8 (16.3%)
*p‐value*		0.92	0.82	0.13	0.13
Diabetes	37 (14.5%)	5 (13.5%)	8 (21.6%)	7 (18.9%)	12 (32.4%)
*p‐value*		0.91	0.09	0.83	0.39
Hypothyroidism	6 (2.4%)	0 (0.0%)	0 (0.0%)	1 (16.7%)	1 (16.7%)
*p‐value*		0.40	0.43	0.71	0.58
History of neuropathy	13 (5.1%)	6 (46.2%)	4 (30.8%)	0 (0.0%)	6 (46.2%)
*p‐value*		0.001	0.049	0.09	0.10
Hepatitis B or C	13 (5.1%)	1 (7.7%)	3 (23.1%)	4 (30.8%)	5 (38.5%)
*p‐value*		0.50	0.26	0.20	0.32

**Table 2:** Risk factors of chemotherapy‐induced peripheral neuropathy in univariate analysis of continuous variables (*n* = 255)

Variable	Overall	Chemotherapy‐induced peripheral neuropathy with each scale used											
		CTCAE‐motor		*p*‐value	CTCAE‐sensory		*p*‐value	WHO item		*p*‐value	Combined scale		*p*‐value
		Yes (*n* = 36)	No (*n* = 219)		Yes (*n* = 33)	No (*n* = 222)		Yes (*n* = 45)	No (*n* = 210)		Yes (*n* = 68)	No (*n* = 187)	
Age	54.2 (9.2)	57.4 (8.3)	53.6 (9.3)	0.02	57.7 (8.3)	53.6 (9.2)	0.02	56.7 (9.2)	53.6 (9.1)	0.04	56.4 (8.9)	53.4 (9.2)	0.02
Cumulative dose													
Oxaliplatin (mg/m^2^)	718.7 (288.5) (*n* = 20)	721.7 (*n* = 1)	718.5 (296.4) (*n* = 19)	0.99	721.8 (*n* = 1)	718.5 (296.4) (*n* = 19)	0.99	862.5 (109.4) (*n* = 4)	682.7 (310.1) (*n* = 16)	0.28	862.5 (109.4) (*n* = 4)	682.7 (310.1) (*n* = 16)	0.28
Carboplatin AUC	27.2 (7.4) (*n* = 66)	28.0 (3.8) (*n* = 7)	27.1 (7.7) (*n* = 59)	0.76	28.3 (4.1) (*n* = 6)	27.1 (7.6) (*n* = 60)	0.70	25.8 (8.9) (*n* = 9)	27.4 (7.2) (*n* = 57)	0.55	26.6 (7.2) (*n* = 15)	27.4 (7.5) (*n* = 51)	0.71
Cisplatin (mg/m^2^)	215.8 (145.9) (*n* = 41)	117.3 (28.3) (*n* = 3)	223.5 (148.8) (*n* = 38)	0.23	126.0 (33.9) (*n* = 2)	220.4 (148.1) (*n* = 39)	0.38	101.0 (1.4) (*n* = 2)	221.7 (147.3) (*n* = 39)	0.26	117.3 (28.3) (*n* = 3)	223.5 (148.7) (*n* = 38)	0.23
Paclitaxel (mg/m^2^)	963.3 (219.7) (*n* = 66)	976.5 (300.0) (*n* = 11)	960.7 (203.5) (*n* = 55)	0.83	984.1 (274.2) (*n* = 13)	958.2 (207.0) (*n* = 53)	0.71	965.2 (294.1) (*n* = 21)	962.4 (178.9) (*n* = 45)	0.96	982.2 (263.9) (*n* = 27)	950.2 (185.8) (*n* = 39)	0.57
Docetaxel (mg/m^2^)	373.9 (75.8) (*n* = 124)	380.3 (74.5) (*n* = 21)	372.6 (76.4) (*n* = 103)	0.67	385.3 (67.9) (*n* = 17)	372.1 (77.1) (*n* = 107)	0.51	401.0 (60.9) (*n* = 18)	369.3 (77.4) (*n* = 106)	0.10	385.4 (68.5) (*n* = 34)	369.5 (78.2) (*n* = 90)	0.30
Alcohol intake	0.2 (0.7)	0.0 (0.0)	0.2 (0.7)	0.10	0.0 (0.0)	0.2 (0.7)	0.12	0.04 (0.3)	0.2 (0.7)	0.16	0.03 (0.2)	0.2 (0.8)	0.04
Fruit intake	1.6 (1.5)	1.7 (0.8)	1.6 (1.6)	0.81	1.5 (0.7)	1.6 (1.6)	0.69	1.6 (0.7)	1.6 (1.6)	0.96	1.6 (0.7)	1.6 (1.7)	0.96
Vegetable intake	1.8 (1.5)	1.7 (0.8)	1.8 (1.6)	0.66	1.6 (0.8)	1.8 (1.6)	0.36	1.7 (0.8)	1.8 (1.6)	0.78	1.7 (0.8)	1.8 (1.7)	0.67
Number of chemotherapy cycles received	6.2 (3.0)	6.5 (3.3)	6.2 (3.0)	0.51	7.2 (3.6)	6.1 (2.9)	0.049	8.3 (4.1)	5.8 (2.5)	<0.001	7.5 (3.7)	5.8 (2.6)	<0.001
Symptom burden (total score)	24.4 (7.0)	26.3 (7.9)	24.0 (6.8)	0.07	27.4 (8.2)	23.9 (6.7)	0.008	26.4 (9.6)	23.9 (6.2)	0.03	26.4 (8.5)	23.6 (6.2)	0.005

Non‐Chinese Asians (primarily of Malay and Indian origin) had higher risk of developing neuropathy than Chinese or Caucasians, only when the WHO scale was used (*p* = 0.01). Also, patients receiving platinum‐based chemotherapy had lower risk of developing CIPN than those receiving taxane‐based chemotherapy (across all CIPN scales used). A particularly high incidence of developing CIPN was observed in patients receiving paclitaxel compared to all other chemotherapy protocols. For many medications in our list, there were not enough incidence of use (i.e., minimum of 5) to allow for further analysis. Metronidazole use had no statistically significant difference (although CIPN incidence was high in this group of patients). Use of statins was implicated in the development of sensory neuropathy and it was also statistically significant variable in the combined CIPN category (*p* = 0.04) and sensory neuropathy item (*p* = 0.03). Diabetes showed a trend (*p* = 0.09) with sensory CIPN only. History of neuropathy was predictive of CIPN when the CTCAE scale was used and showed trends when the WHO scale was used. Hepatitis was not a statistically significant risk factor, although CIPN incidence was high in this group of patients. Detailed data are presented in Table 1.

Table 2 shows statistically significant predictors in continuous variables. Consistently (older) age and number of chemotherapy cycles received were significant risk factors. Alcohol intake was significant only when the combined scale was used. Symptom burden (mean of all symptoms from the EORTC scale) was also linked with CIPN in this univariate analysis, alongside a number of symptoms in at least the combined CIPN scale. These included pain interfering with daily activities (*p* = 0.02), trouble sleeping (*p* = 0.04), being tired (*p* = 0.01), appetite loss (*p* = 0.04), constipation (*p* = 0.001), worrying (*p* = 0.054), and difficulty remembering (*p* = 0.01). Fruit and vegetable intake were not linked with CIPN. A stepwise logistic regression just for the individual symptoms showed that two symptoms were linked with the higher risk of CIPN, namely difficulty remembering (OR = 1.61, *p* < 0.05; 95% CI = 1.10–2.34) and constipation (OR = 2.06, *p* < 0.01; 95% CI = 1.29–3.29).

The final multivariate logistic regression model (Table 3) of all univariate predictors with *p*‐value < 0.20 observed in the previous analyses showed that patients receiving platinum‐based chemotherapy had lower risk of CIPN compared to those receiving taxane‐based chemotherapy; those with history of neuropathy had higher risk for (motor) CIPN, as well as older patients. Symptom burden had some contribution to (primarily to sensory) CIPN. Number of chemotherapy cycles received was also a strong predictor of CIPN. One unit of alcohol use decreased the risk of CIPN by 68% (only in the combined scale).

**Table 3:** Logistic regression model of chemotherapy induced peripheral neuropathy‐related risk factors for each assessment scale used

CTCAE‐motor scale		CTCAE‐sensory scale		WHO item		Combined CIPN scale	
Variable	OR (95% CI)	Variable	OR (95% CI)	Variable	OR (95% CI)	Variable	OR (95% CI)
Chemotherapy group		Chemotherapy group		Race		Chemotherapy group	
Taxanes	Ref	Taxanes	Ref	Chinese	Ref	Taxanes	Ref
Platinum	0.27* (0.07, 0.91)	Platinum	0.27 (0.07, 1.07)	Non‐Chinese Asians	1.27 (0.35, 4.68)	Platinum	0.20** (0.07, 0.58)
Combined	0.46 (0.18, 1.21)	Combined	0.38 (0.14, 1.06)	Caucasian	0.23 (0.03, 1.59)	Combined	0.51 (0.24, 1.08)
Female	1.72 (0.43, 6.95)	Female	2.36 (0.52, 10.75)	Chemotherapy group		Female	1.01 (0.34, 3.04)
History of neuropathy	8.36** (1.74, 40.13)	History of neuropathy	2.49 (0.59, 10.46)	Taxanes	Ref	Smoking history	
Age	1.02* (1.02, 1.12)	Age	1.08** (1.03, 1.13)	Platinum	0.25* (0.08, 0.79)	Never	Ref
Symptom burden	1.04 (0.99, 1.09)	Number of chemotherapy cycles received	1.12 (0.98, 1.29)	Combined	0.50 (0.20, 1.28)	Current	1.53 (0.26, 9.13)
		Symptom burden	1.06* (1.01, 1.11)	Smoking history		Ex‐smoker	0.86 (0.31, 2.34)
				Never	Ref	History of neuropathy	2.15 (0.56, 8.31)
				Current	2.54 (0.42, 15.17)	Age	1.06** (1.02, 1.10)
				Ex‐smoker	1.18 (0.36, 3.89)	Alcohol intake	0.32* (0.12, 0.86)
				Hepatitis B or C	3.93 (0.92, 16.83)	Number of chemotherapy cycles received	1.19** (1.07, 1.32)
				Age	1.06* (1.01, 1.11)	Symptom burden	1.06* (1.01, 1.11)
				Alcohol intake	0.30 (0.07, 1.02)		
				Number of chemotherapy cycles received	1.24** (1.07, 1.43)		
				Symptom burden	1.05 (0.995, 1.11)		

Note

Each regression model is presented in one column, the dependent variable is shown in the first row of the table and all variables were put in the regression as independent variables

* *p* < 0.05.

** *p* < 0.01.

## 7 DISCUSSION

This study assessed CIPN clinical risk factors using a prospective design and a wide range of potential predictors. Overall CIPN incidence was lower in this study than that reported in the literature, and this has to do probably with the scales used; past studies have used quality of life scales to estimate CIPN, which often include a range of general/broader items to indicate neuropathy. Also, clinician‐based assessments, such as the NCI‐CTCAE tend to underestimate CIPN incidence (Dorsey et al., 2019). We have explained these reasons in more detail in the parent larger study (Molassiotis et al., 2019). However, in a systematic review it was shown that CIPN incidence at 6 months was 30% (Seretny et al., 2014) and our incidence in the combined tools was 26%. Key risk factors identified include older age, history of neuropathy, symptom burden, alcohol intake (cautiously accepted as a risk factor in this study due to the small number of events needing further clarification in the future) and number of chemotherapy cycles used. Patients receiving platinum‐based chemotherapy had 17%–27% less chance of developing CIPN compared to those receiving taxane‐based chemotheraopy. Risk factors were not always consistent across the scales used. This may reflect sensitivity or reliability issues with the various scales measuring CIPN. As the measurement tool(s) used in future risk factor research will be related with the identification of specific risk factors, it is important to use the most reliable and valid CIPN scale (Cavaletti et al., 2013; Dorsey et al., 2019) or a combination of scales to maximize the “pick up” rates of these tools that will include both patient‐reported outcomes and objective CIPN indicators, such as with the Total Neuropathy Score clinical version (TNSc) (McCrary et al., 2017).

Older age somewhat contributed to CIPN (6% more chances), supporting findings from past research (Bandos et al., 2018; Hershman et al., 2016; Miaskowski et al., 2017). History of neuropathy was a potential risk factor for motor neuropathy and its ORs were high in the other CIPN scales (but did not reach statistical significance). History of neuropathy was mainly linked with motor CIPN, with patients having such history being more than eight times at a higher risk for developing motor CIPN. The limited research of the past does not differentiate the role of this variable in the type of neuropathy, hence this is a novel finding. Statin use as a risk factor for CIPN is also another novel finding of this study, although this finding from univariate analysis was not sustained in the final model, likely because of the small number of patients receiving statins in this sample. This finding supports an earlier case‐control study on patients receiving statins, although the sample in the latter study was not focusing on cancer patients (Gaist et al., 2002). However, more recent work from a case‐control study showed that ever use of statins was not associated with a higher risk of polyneuropathy (Svendsen et al., 2017). This finding needs further elaboration in the future, although if a risk exists, it is probably minimal. Metronidazole use as a potential risk factor should also be investigated in the future, as the incidence of CIPN in this subgroup was high (up to 41.7%) and literature suggests sensory and autonomic neuropathy as a result of such use (Hobson‐Webb, Roach, & Donofrio, 2006), although the small number of such cases may have contributed to the nonsignificant results shown. Such future work should clearly delineate duration of use, dose and timing of use, which were not collected in our study and hence pose limitations in interpreting this result.

Symptom burden is a new variable implicated in the development of CIPN. Whether this finding is attributed to collinearity with CIPN or symptom burden influencing the development (and/or severity) of CIPN is not yet clear. Two particular symptoms (out of 18 assessed) had the strongest relationship with CIPN, including constipation and difficulty remembering. It may be that neuronal damage related to CIPN leads to constipation or cognitive deficits in patients. The link between autonomic neuropathy and constipation may be the result of neurogenic bowel/disautonomia or constipation may be one of the indications of constituent autonomic neuropathy. CIPN and cognitive changes such as difficulty remembering/”chemofog” may be link as a result of neuroinflammation postchemotherapy, which has been discussed as a potential mechanism for behavioral toxicities (Vichaya et al., 2015). It will be interesting to explore these assumptions in the future more concretely and have a more in‐depth understanding of the link between symptoms/symptom burden and CIPN. This finding is further supported by recent research showing that patients with CIPN had significantly poorer functional status (Miaskowski et al., 2017).

The role of (chronic) alcohol use in the development of CIPN is less clear, as contradictory findings have been presented in the literature, probably due to the inherent problems in measuring alcohol use accurately. Our findings suggest that no alcohol use had some protective effect in CIPN, but this was not consistent across all the scales used. Also, our sample had very few heavy drinkers and this may have impacted on the results. Alcohol use (as well as diabetes) may be associated with the development of neuropathy before the chemotherapy, and we have seen that preexisting neuropathy was a key CIPN risk factor.

The number of chemotherapy cycles received was a strong predictor both in univariate and multivariate analyses. This is not linked with cumulative dose (as the latter was not shown to be predictive of CIPN in our study). Hence, this finding may imply that “time” after starting chemotherapy may be strongly linked with the development of CIPN, suggesting that CIPN is time‐dependent rather than dose‐dependent, although the link between cumulative dose and CIPN has been reported in past literature but not consistently (Seretny et al., 2014).

Two parameters in the final predictive model need some more consideration in the development of CIPN. Firstly, the role of hepatitis (possibly as a result of taking neurotoxic antiviral agents in the past or even as a result of disturbance in the pharmacokinetics of the chemotherapy drugs, i.e., decreased liver function and/or increased drug exposition). Secondly, current smoking with perhaps its connection with pain pathways. Both of them had very high odds ratios (3.93 and 1.18–2.54, respectively) but both these ORs were not statistically significant, highly likely as a result of the small number of patients reporting these two variables (*n* = 13 and 7, respectively). Future research should provide more insight about the potential risk for CIPN for hepatitis and smoking status.

Some variables in the study had small frequency counts, and this may affect the interpretation and generalizability of the results and should be perceived as preliminary only. Identification of risk factors may assist the clinician to make chemotherapy treatment decisions accordingly in order to minimize not only the development of CIPN but also the morbidity and health care utilization linked with higher incidence of CIPN (while clinical effectiveness is not compromized). However, the state of science in this area is not yet optimal for such clinical decisions, and more research in elucidating strong CIPN‐related risk factors is needed, including the development of predictive models. Other consistent risk factors, such as higher BMI and obesity were not assessed in this study and these should be included in future models.

This study confirms the role of (older) age; number of chemotherapy cycles received, and type of chemotherapy as key CIPN risk factors. The role of past neuropathic damage specifically linked with motor CIPN and (chronic) alcohol consumption are also important new variables to consider alongside the presence of symptom burden/specific symptoms that may form a symptom cluster around neuropathy. Risk factor knowledge can assist health professionals in educating patients in a more targeted way about this symptom experience and introduce more regular assessment of CIPN particularly in those at higher risk, in order to monitor its development and the impact it may have on patients’ quality of life. Preventive interventions may need to be initiated to those with high risk of CIPN.

## CONFLICT OF INTEREST

The authors hereby certify that we have all seen and approved this manuscript. We guarantee that the paper is the authors’ original work and that it has not been the subject of prior publication and is not under consideration for publication elsewhere. On behalf of all the co‐authors, the corresponding author bears full responsibility for the submission. There are no financial or other relationships that might pose a conflict of interest.

## Data Availability

The data that support the findings of this study are available from the corresponding author upon reasonable request.
